# Practice effects in cognitive assessments three years later in non-carriers but not in symptom-free mutation carriers of autosomal-dominant Alzheimer's disease: Exemplifying procedural learning and memory?

**DOI:** 10.3389/fnagi.2022.905329

**Published:** 2022-10-05

**Authors:** Ove Almkvist, Caroline Graff

**Affiliations:** ^1^Divisions of Clinical Geriatrics, Department of Neurobiology Care Sciences and Society, Karolinska Institutet, Stockholm, Sweden; ^2^Theme Inflammation and Aging, Karolinska University Hospital, Stockholm, Sweden; ^3^Department of Psychology, Stockholm University, Stockholm, Sweden; ^4^Divisions of Neurogeriatrics, Department of Neurobiology Care Sciences and Society, Karolinska Institutet, Stockholm, Sweden

**Keywords:** practice effect, cognition, Alzheimer's disease, autosomal-dominant, normal ageing, progression

## Abstract

Practice effects (PEs) defined as an improvement of performance in cognition due to repeated assessments between sessions are well known in unimpaired individuals, while less is known about impaired cognition and particularly in latent brain disease as autosomal-dominant Alzheimer's disease. The purpose was to evaluate the general (across tests/domains) and domain-specific PE calculated as the annual rate of change (ARC) in relation to years to the estimated disease onset (YECO) and in four groups of AD: asymptomatic mutation carriers (aAD, *n* = 19), prodromal, i.e., symptomatic mutation carriers, criteria for AD diagnosis not fulfilled (pAD, *n* = 4) and mutation carriers diagnosed with AD (dAD, *n* = 6) as well as mutation non-carriers from the AD families serving as a healthy comparison group (HC, *n* = 35). Cognition was assessed at baseline and follow-up about 3 years later by 12 tests covering six domains. The aAD and HC groups were comparable at baseline in demographic characteristics (age, gender, and education), when they were in their early forties, while the pAD and dAD groups were older and cognitively impaired. The results on mean ARC for the four groups were significantly different, small, positive, and age-insensitive in the HC group, while ARC was negative and declined with time/disease advancement in AD. The differences between HC and aAD groups in mean ARC and domain-specific ARC were not significant, indicating a subtle PE in aAD in the early preclinical stage of AD. In the symptomatic stages of AD, there was no PE probably due to cognitive disease-related progression. PEs were the largest in the verbal domain in both the HC and aAD groups, indicating a relationship with cognitive vulnerability. The group-related difference in mean ARC was predominant in timekeeping tests. To conclude, the practice effect in over 3 years was suggested to be linked to procedural learning and memory.

## Introduction

The practice or retest or learning effect refers to a phenomenon that individuals, who are assessed a second time (not within the same session) with the same neuropsychological test(s), show improved performance in the absence of an intervention. The practice effect (PE) occurs both in normal individuals (Calamia et al., 2012; Machulda et al., 2013, 2017; Gross et al., 2015; Jutten et al., 2020; Samaroo et al., 2020; Lim et al., 2021) and in patients diagnosed with cognitive impairment (Machulda et al., 2013; Gross et al., 2018; Jutten et al., 2020). The occurrence of PE is so common that the absence of PE is considered a potential marker of disease progression (Zehnder et al., 2007; Hassenstab et al., 2015; Elman et al., 2018; Jutten et al., 2020; De Simone et al., 2021) and disease (Cooper et al., 2004; Zehnder et al., 2007). The common knowledge of PE is presented and summarized in large meta-analyses (Calamia et al., 2012; Duff and Hammers, 2020; Jutten et al., 2020).

There are a number of core issues regarding PE. The size has been estimated to be 0.2–0.6 standard deviations in normal individuals (Van der Elst et al., 2008) although smaller and larger estimates have been reported (Bartels et al., 2010; Scharfen et al., 2018a; Duff and Hammers, 2020). The size of the effect may vary with cognitive domain and the specific test (Calamia et al., 2012; Salthouse, 2015; Gross et al., 2018; Samaroo et al., 2020), premorbid/baseline level of cognitive function (Bartels et al., 2010; Arendasy and Sommer, 2017; Scharfen et al., 2019), test experience (Salthouse, 2015), task requirement (Arendasy and Sommer, 2017; Scharfen et al., 2018b), personality, e.g., anxiety (Jendryczko et al., 2019), length of retest intervals (Falleti et al., 2006; Calamia et al., 2012; Machulda et al., 2013; Salthouse, 2015; Scharfen et al., 2018b; Jutten et al., 2020), retest interval conditions, e.g., treatment (Jacobs et al., 2017; Jutten et al., 2020; Wang et al., 2020), demographic characteristics such as age (Salthouse, 2010; Calamia et al., 2012) and education (Bartels et al., 2010), type and severity of disease ranging from dementia (Cooper et al., 2004; Gross et al., 2015, 2018; Sánchez-Benavides et al., 2016), to mild cognitive impairment (Cooper et al., 2004; Bläsi et al., 2009; Calamia et al., 2012; Duff and Hammers, 2020), presence of comorbidity and risk factor for cognitive decline like APOE status and AD biomarkers (Zehnder et al., 2007; Machulda et al., 2013; Oltra-Cucarella et al., 2018; Jutten et al., 2020; Lim et al., 2021), and relationship with brain findings (Duff et al., 2017, 2018; Wilson et al., 2018; Jutten et al., 2020; Samaroo et al., 2020). Although there is a lot of knowledge regarding PE, there is still incomplete knowledge of serial assessments (Ivnik et al., 2000; Bartels et al., 2010; Heilbronner et al., 2010; Wilson et al., 2018; Scharfen et al., 2019; Jutten et al., 2020; Samaroo et al., 2020; Lim et al., 2021) and particularly on PE in asymptomatic latent disease in the preclinical stage of autosomal-dominant AD (adAD).

The purpose of the study was to investigate PE in repeated assessments of cognitive functions in carriers and non-carriers from six families with adAD. These individuals could be divided into four groups associated with varying degrees of present cognitive impairment: mutation carriers diagnosed with clinical dementia of AD (dAD), mutation carriers with symptoms but unfulfilled diagnostic criteria of AD, i.e., prodromal AD (pAD) and mutation carriers lacking symptoms, i.e., asymptomatic AD (aAD), who will develop Alzheimer's dementia in future, and finally non-carriers from adAD families serving as a healthy comparison group (HC). These individuals were followed with repeated clinical examinations including cognitive assessment of performance in five domains. These domains are selectively sensitive to brain involvement in AD; episodic memory is considered most sensitive and affected early in the disease course, while verbal knowledge is considered relatively stable and affected relatively late in the disease course.

In adAD, there is an option to characterize each individual in terms of disease advancement, i.e., years to estimated clinical onset (YECO; Bateman et al., 2012; Almkvist et al., 2017).

Following this outline, the first aim was to investigate the degree of PE measured as the annual rate of change (ARC) between two assessments in the four groups of AD participants (dAD, pAD, aAD, and HC). The hypothesis was that groups differed in relation to stage of disease progression showing PE in HC and possibly in aAD followed by the absence of PE in pAD and dAD. The second aim was to compare PE in specific cognitive domains/tests in HC and AD. The hypothesis was that PE varies between cognitive domains in relation to regional brain involvement linked to brain vulnerability in AD and aging. The third aim was to identify when PE is observed, or conversely when PE is not observed in disease progression in mutation carriers. The hypothesis was that PE is inversely associated with disease progression (YECO) in mutation carriers and relatively unrelated to age in non-carriers (YECO).

## Materials and methods

### Participants

Adult members of six families carrying an early onset AD mutation were invited to a comprehensive clinical examination at the Memory Clinic, Karolinska University Hospital Huddinge, Sweden. Ninety-four individuals accepted to participate in the baseline examination and most individuals accepted follow-up examination (*n* = 64). There was no significant difference between the 94 and the 64 individuals in demographics (age, gender, and years of education), cognitive screening (MMSE), or mutation status (carrier/non-carrier) (all *p*-values of >0.1). The study concerned 29 mutation carriers from six adAD families and 35 non-carriers from the same six families.

Three families carried an APP mutation the Swedish *APP* K670N/M671L (Axelman et al., 1994), or the Arctic *APP* E693G mutation (Nilsberth et al., 2001), or the London *APP* V717I mutation (Goate et al., 1991). Three families carried a *PSEN1* I143T mutation (Keller et al., 2010); or the M146V mutation (Haltia et al., 1994); or the H163Y mutation (Axelman et al., 1998).

In autosomal-dominant AD families, it is possible to estimate each individual's time (years) to the expected clinical onset (YECO) of symptoms based on information from previous mutation carriers in each family. The family-specific mean age at onset of clinical symptoms is 36 ± 2 years for *PSEN1* I143T (Keller et al., 2010), 36 ± 3 years for *PSEN1* M146V (Haltia et al., 1994), 51 ± 7 years for *PSEN1* H163Y (Axelman et al., 1998; Thordardottir et al., 2015), 54 ± 5 years for *APP*_SWE_ (Axelman et al., 1994; Thordardottir et al., 2015), 56 ± 3 years for *APP*_ARC_ (Nilsberth et al., 2001; Thordardottir et al., 2015), and 57 ± 5 years for London *APP* V717I (Goate et al., 1991). For each participant, both mutation carriers and non-carriers, YECO was calculated as the difference between the individual's age at the time of the examination minus the family-specific age at clinical onset, i.e., YECO = the individual's present age—the expected family-specific onset of symptoms.

### Procedure

All participants, mutation carriers and non-carriers, had a comprehensive clinical examination at each visit, which included somatic, neurological, psychiatric status, cognitive screening with the Mini-Mental Status Examination (MMSE; Folstein et al., 1975) and assessment of cognitive functions (see below), sampling of blood, urine and cerebrospinal fluid for standard analyses, and magnetic resonance imaging of brain anatomy. Although clinical examinations started as far back as 1993, essentially the same protocol was followed throughout the study.

### Diagnosis

Based on the clinical examination at baseline, six mutation carriers were diagnosed as having dementia according to the Diagnostic and Statistical Manual of Mental Disorders (DSM-IV) (American Psychiatric Association, 1994) and AD according to the Alzheimer's Disease and Related Disorders Association (NINCDS-ARDRA) criteria (McKhann et al., 1984). These individuals constitute the dAD group. Mild Cognitive Impairment (MCI) was diagnosed following revised Petersen criteria (Winblad et al., 2004) and four mutation carriers were diagnosed as having MCI but criteria for AD were not fulfilled; they constitute the prodromal AD group. The 19 non-diagnosed mutation carriers had no AD-related symptoms and were cognitively unimpaired and considered to be asymptomatic although they were mutation carriers; they constitute the asymptomatic AD group.

At the first follow-up examination about 3 years after the baseline examination, 10 mutation carriers were diagnosed with AD (three pAD and one aAD at baseline developed dementia at follow-up), two mutation carriers were diagnosed as prodromal at follow-up, i.e., symptomatic, but AD criteria were not fulfilled (one aAD at baseline changed into pAD and one pAD remained as pAD). Seventeen mutation carriers were still evaluated as asymptomatic at follow-up. All individuals in the HC group were healthy and cognitively unimpaired. One healthy non-carrier had lifelong selective non-progressive cognitive difficulties due to a specific syndrome (topographical disorientation); the data for this participant were retained in the study but excluded for selectively impaired tests caused by the specific syndrome. Another non-carrier had been a boxer and participated in tournaments in young adulthood and later he had been affected by multiple small brain infarcts in middle age, which motivated to exclude him from the study.

### Procedure

All individuals went through a standard comprehensive clinical examination, which included an interview with the participant and often with a close informant. The examination included somatic, neurological, and psychiatric statuses, sampling of blood, and cerebrospinal fluid [(CSF); (beta-amyloid, total, and phosphorylated tau)], brain imaging using magnetic resonance imaging (e.g., global atrophy); and electroencephalography examination, and assessment of cognitive function (see below). The same protocol has been followed throughout the study during follow-up visits.

### Assessment of cognitive function

Premorbid global cognitive function was assessed based on demographic information and reading test results (Tallberg et al., 2006). The following tests were used to assess cognitive domains: the Information and Similarities tests from the Wechsler Adult Intelligence Scale-Revised (Wechsler, 1981; Bartfai et al., 1994; WAIS-R) for verbal ability, the Block Design from WAIS-R and the Rey–Osterrieth Copy tests (Lezak et al., 2004) for visuospatial ability, the Digit Span from WAIS-R and the Corsi Span (Lezak et al., 2004) for short-term memory (STM), the Rey Auditory Verbal Learning test, including learning and retention after 30 min, and the Rey–Osterrieth retention after 30 min (Lezak et al., 2004) for verbal and visuospatial episodic memory, the Trail Making A test (Lezak et al., 2004) for attention and the Digit Symbol from WAIS-R and the Trail Making B (Lezak et al., 2004) for executive function. Raw scores were converted to *z*-scores using a reference group of healthy adults (Bergman et al., 2007). The z-scores are always directed so that positive values indicate a favorable performance.

### Practice effect

The main outcome measure was the annual rate of change (ARC) defined as the unweighted score of the test result in z-score at the second visit—test result in z-score at the first visit divided by the time interval in years (one decimal) between the first and second visits for each of the 12 tests. Unweighted ARC score was computed for each domain; verbal (Information and Similarities, visuospatial (Block Design and Rey–Osterrieth Copy), STM (Digit Span and Corsi Span), episodic memory (RAVL learning and retention and Rey–Osterrieth retention), attention (TMTA), and finally executive (Digit Symbol and TMTB). Missing data occurred infrequently (total number of observations = 12 tests × 2 visits × 64 participants = 1,536, number of missing data = 92, 6.0%, half of the missing data occurred in RAVL retention due to inability, recorded as missing and not as 0).

The follow-up examination occurred after about 3 years (M±SD: 3.0 ± 3.5, range 0.6–20 years). Most participants had retest intervals between 2 and 4 years. The few extremely short and long retest intervals were due to participants' personal conditions.

### Statistical analyses

Descriptive statistics were used for background characteristics. Bar graphs and scatter plots were used to visualize the results. A one-sample *t*-test was used to analyze if ARC deviated from 0. A one-way ANOVA was used to analyze group differences on ARC. A multivariate ANOVA was used to analyze the main effects of group and domain as well as the group-by-domain interaction on ARC.

## Results

The background characteristics of participants in the four groups at the baseline visit are shown in Table 1. There was no significant difference between groups in age, gender, years of education, retest interval, premorbid IQ, and the number of *APOE* e4 alleles (all *p*-values of >0.1), while groups differed significantly in YECO (*F* = 4.84, df=3/59, *p* < 0.01, η^2^ = 0.20) and global cognition assessed by MMSE (*F* = 17.96, df = 3/42, *p* < 0.001, η^2^ = 0.56) in relation to the progression of AD.

**Table 1:** Background characteristics at baseline in non-carriers (Healthy Comparison group, HC) and mutation carriers with AD (asymptomatic, prodromal and diagnosed AD).

	**Non-carriers**	**Mutation carriers**		
	**HC**	**Asymptomatic**	**Prodromal**	**Diagnosed AD**
*N* (females/males)	35 (17/18)	19 (6/13)	4 (1/3)	6 (2/4)
Age, y	39.7 ± 12.9	37.8 ± 10.1	51.3 ± 7.1	49.6 ± 7.1
Range, y	17–62	21–53	41–57	40–56
Education, y	11.0 ± 2.3	11.8 ± 2.1	12.5 ± 3.1	9.7 ± 1.8
Range, y	7–18	9–16	10–17	7–12
YECO at 1st visit, y	−9.5 ± 6.7	−12.8 ± 8.1	−0.1 ± 2.3	+0.6 ± 5.5
Range	−27 to +10	−26 to −3	−4 to +1	−6 to +6
Retest interval, y	3.4 ± 2.4	3.0 ± 1.9	3.3 ± 2.9	1.9 ± 0.8
Range, y	1–11	1–20	1–8	1–3
Premorbid IQ, iq-score	104 ± 7.7	110 ± 8.4	108 ± 9.8	111 ± 8.3
Range	91–116	94–123	97–116	97–111
MMSE, score	29.0 ± 1.6	28.8 ± 1.7	26.8 ± 1.5	21.0 ± 5.3
Range, score	23–30	27–30	24–28	14–26
APOE e4, frequency	10/35	7/19	0/4	2/6

The cognitive test results at baseline in each test for the HC and AD (aAD, pAD, and dAD) groups are shown in Supplementary Table 1. The groups differed significantly in 10 of the 12 tests and most strongly in episodic memory (RAVL learning, RAVL retention, and Rey– Osterrieth retention), executive function (Digit Symbol and TMTB), and visuospatial performance (Block Design) (see Table 2). The HC and aAD groups did not differ significantly in any test (all *p*-values of >0.1). The aAD and pAD groups differed significantly in two tests: TMTA (*t* = 2.60, df = 21, *p* < 0.05, Cohen's *d* = 1.31) and TMTB (*t* = 3.50, df = 19, *p* < 0.01, Cohen's *d* = 1.94). The pAD and dAD groups did not differ significantly in any test (all *p*-values of >0.1), although the mean *z*-scores were much poorer in the dAD group compared to the pAD group.

**Table 2:** Practice effects expressed as the Annual Rate of Change (ARC) across cognitive domains at baseline in non-carriers (Healthy Comparisons group, HC) and mutation carriers varying in stage of AD disease course (asymptomatic AD and combined prodromal AD and dementia AD).

	**Non-carriers**	**Mutation carriers**			
**Domain**	**HC**	**aAD**	**pAD and dAD**	***P***	**η^2^**
Mean cognition	+0.05 ± 0.11	+0.01 ± 0.17	−0.35 ± 0.33	***	0.40
Verbal	+0.19 ± 0.36	+0.10 ± 0.17	−0.13 ± 0.36	**	0.15
Visuospatial	+0.06 ± 0.34	−0.16 ± 0.68	−0.50 ± 0.49	*	0.11
STM	−0.02 ± 0.37	−0.02 ± 0.14	−0.26 ± 0.31	*	0.12
Episodic memory	+0.08 ± 0.26	−0.01 ± 0.16	−0.16 ± 0.28	Ns	0.08
Executive function	+0.04 ± 0.28	−0.05 ± 0.12	−0.41 ± 0.62	***	0.23
Attention	−0.02 ± 0.40	+0.04 ± 0.28	−0.37 ± 1.04	**	0.17

Significance and eta-square (η^2^) from one-way (group) ANOVA on each domain.

ns, not significant; ^*^p < 0.05, ^**^p < 0.01, ^***^p < 0.001.

I. PE across cognitive tests in AD groups (aAD, pAD, and dAD) in comparison to HC

**Figure 1:**
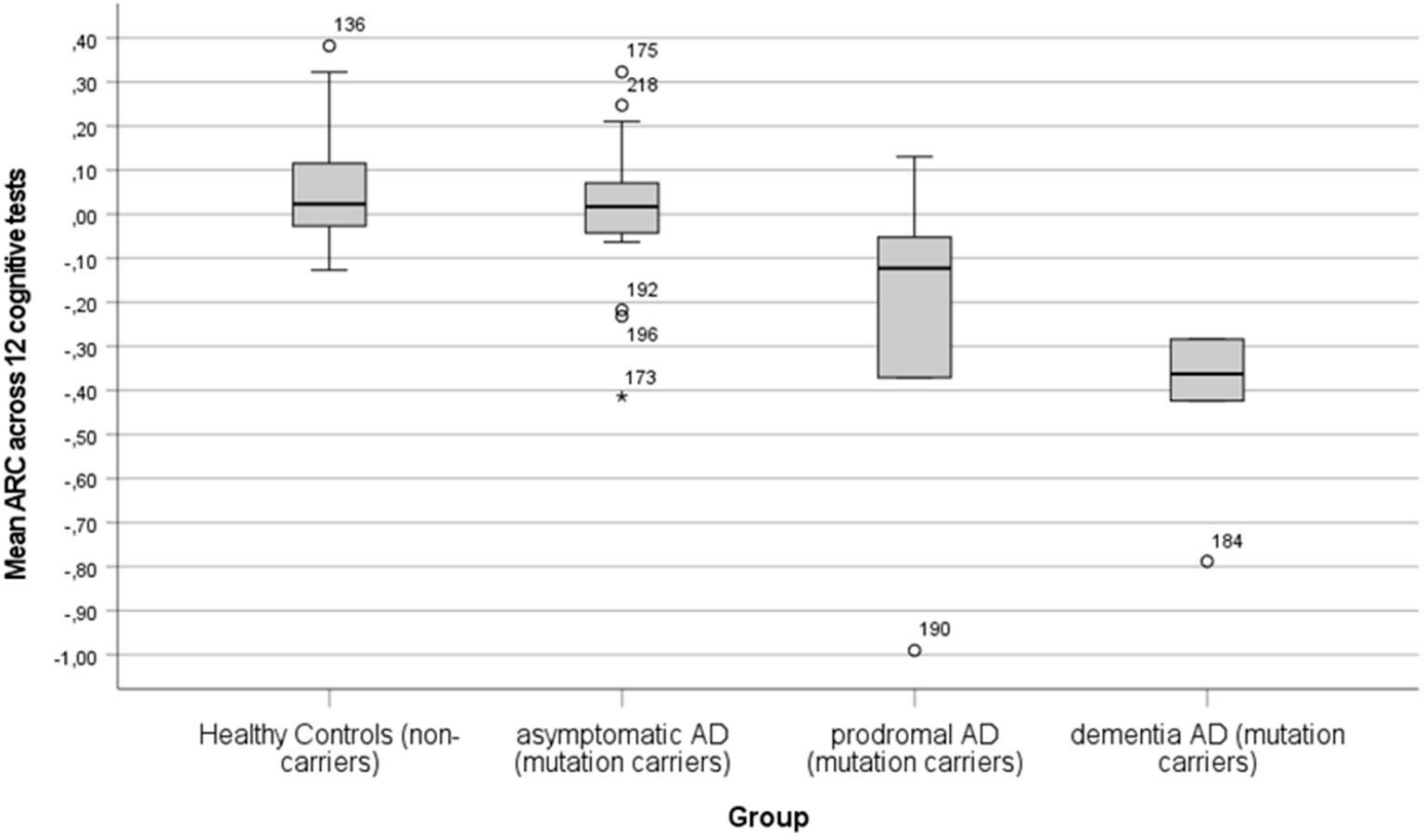
A bar graph showing the mean annual rate of change (ARC) with error bars in HC (non-carriers), aAD (asymptomatic mutation carriers), pAD (symptomatic mutation carriers, AD diagnosis nor fulfilled), and dAD (mutation carriers with AD diagnosis).

The practice effect was evaluated by the mean ARC in the 12 cognitive tests for the AD (aAD, pAD, and dAD) and HC groups. In Figure 1, a bar graph shows the mean ARC for the four groups. The hypothesis that the mean ARC equals 0 was rejected for the HC (*t* = 2.89, df = 34, *p* < 0.01, Cohen's *d* = 0.49) and dAD (*t* = 4.57, df = 4, *p* < 0.01, Cohen's *d* = 2.04) groups, but not for the aAD and pAD groups (*p*-value of >0.1). The mean ARC index differentiated the groups significantly (*F* = 14.59, df = 3/63, *p* < 0.001, η^2^ = 0.88). The difference in mean ARC between the HC (M±SD: 0.05 ± 0.12) and aAD (M±SD: 0.01 ± 0.17) groups was not significant (*p* > 0.1), while the difference in mean ARC between the aAD (M±SD: 0.01 ± 0.17) and pAD (M±SD: −0.28 ± 0.44) groups was significant (*t* = 2.37, df = 22, *p* < 0.05, Cohen's *d* = 1.19). The difference in ARC between the pAD (M±SD: −0.28 ± 0.44) and dAD (M±SD: −0.42 ± 0.21) was not significant (*p* > 0.1).

II. PE in cognitive tests/domains in HC and AD (aAD, pAD, and dAD) groups

The practice effect was evaluated by means of ARC in each cognitive test for the HC and AD groups; the descriptive data are shown in Supplementary Table 2. The four groups were significantly differentiated in 8 of the 12 tests. The practice effect was strongest in three tests, in which performance was measured by timekeeping (Digit Symbol, TMTA, and TMTB). The size of PE in the HC group varied between tests from the largest in the Similarities test (*z* = +0.23) followed by Information (*z* = +0.15) and RAVL learning and Rey–Osterrieth retention (*z* = +0.11) and Block Design (*z* = 0.08) and small in four tests (Digit Span, RAVL retention, Digit Symbol, and TMTB). Unexpectedly, the PE was negative in three tests (Rey–Osterrieth Copy, Corsi Span, and TMTA). The pairwise group differences were not significant in any test for the HC vs. aAD groups and the pAD vs. dAD groups (all *p-*values of >0.1) probably due to small sample sizes.

To increase the sample size in groups, the 12 test results were aggregated into six *a priori* cognitive domains: verbal (Information and Similarities), visuospatial (Block Design and Rey–Osterrieth Copy), STM (Digit Span and Corsi Span), episodic memory (RAVL learning, RAVL retention, and Rey–Osterrieth retention), executive function (Digit Symbol and TMTB), and attention (TMTA). The main outcome of a multivariate analysis (MANOVA) with domain as within independent factor and group as between factor on ARC as dependent factor showed that the group effect was significant (*F* = 7.14, df = 3/55, *p* < 0.001, η^2^ = 0.28), while the domain, as well as the group-by-domain interaction effects, were not significant (*p*-value of >0.1).

Still, the sample size was small in the pAD and dAD groups, so these groups were combined into a symptomatic AD (sAD) group encompassing mild and moderate cognitive impairment. The domain-specific ARC data for the three groups and the six cognitive domains are shown in Table 2. The group effect was significant in five of the six domains (*F* = 10.89, df = 2/56, *p* < 0.001, η^2^ = 0.28). The domain effect was not significant (*p* = 0.08), and the group-by-domain interaction was not significant (*p* > 0.1). The addition of APOE e4 and/or education as covariates did not influence the outcome (*p*-value of >0.1).

The largest PE in the HC group was seen in the verbal domain (*z* = +0.19), and this was statistically different from 0 (*p* < 0.01). In the aAD group, PE was largest in the verbal domain (*z* = +0.09, *p* < 0.05). In the sAD group, some retest changes were negative and significant: visuospatial (*z* = −0.39, *p* < 0.05), STM (*z* = −0.33, *p* < 0.05), and executive (*z* = −0.44, *p* < 0.05).

III. PE in relation to disease advancement in HC and AD (aAD, pAD, and dAD) groups

**Figure 2:**
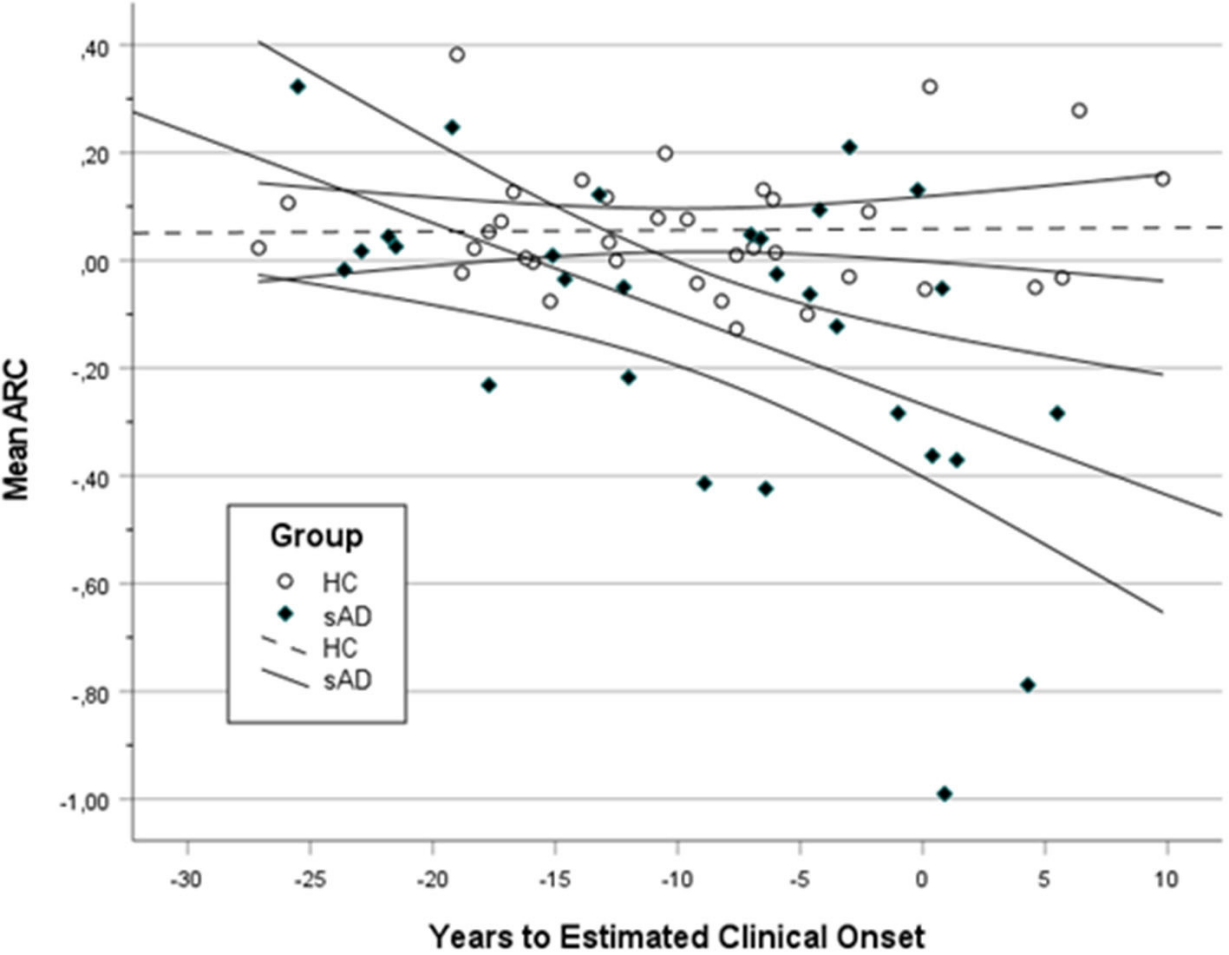
A scatter plot showing the mean annual rate of change (ARC) in HC and all AD (aAD, pAD, and dAD) in relation to years to estimated clinical onset (YECO) with a 95% confidence interval surrounding the linear regression line.

The relationship between PE and time of disease progression (YECO) was analyzed including all participants. It was hypothesized that PE is relatively stable in healthy individuals but varies with the degree of cognitive impairment and finally disappears in AD according to previous research. In Figure 2, a scatter plot is presented showing the mean ARC in relation to the time of disease advancement (YECO) for all participants divided into two groups, HC vs. AD. The graph visualized the regression line and the 95% confidence interval for the two groups. The regression for the HC group was linear and practically invariant in relation to time (*r* = 0.02). The equation for the HC group was ARC = 0.058 + 0.000 ^x^ YECO, i.e., PE = 0.058. The regression for the AD group (combining the aAD, pAD, and dAD into one AD group) was best described by a linear equation that was significant with YECO as a single predictor (*r* = 0.53, *F* = 10.54, df = 1/27, *p* < 0.05, *r*^2^ = 0.28); the equation runs as follows: mean ARC = −0.267 – 0.530 × YECO. The intersection between the HC and AD groups occurred at YECO~ −20, i.e., about 20 years before the estimated clinical onset. Looking at the intersection of confidence intervals, the HC and AD groups were separated at YECO~ −12. Compared to the linear model, a curvilinear model was less powerful as well as models, in which other possible predictors (APOE e4 and/or years of education) were added. The alternative models did not increase the explanatory power.

Looking at Figure 2, a number of individuals both in the HC and AD groups were obvious outliers. In the HC group, three individuals had high positive ARC values (>0.30). In the AD group, there were at least three positive outliers (ARC > 0 and YECO > −4 close to the estimated onset) and five negative outliers far below the lower confidence line.

**Figure 3:**
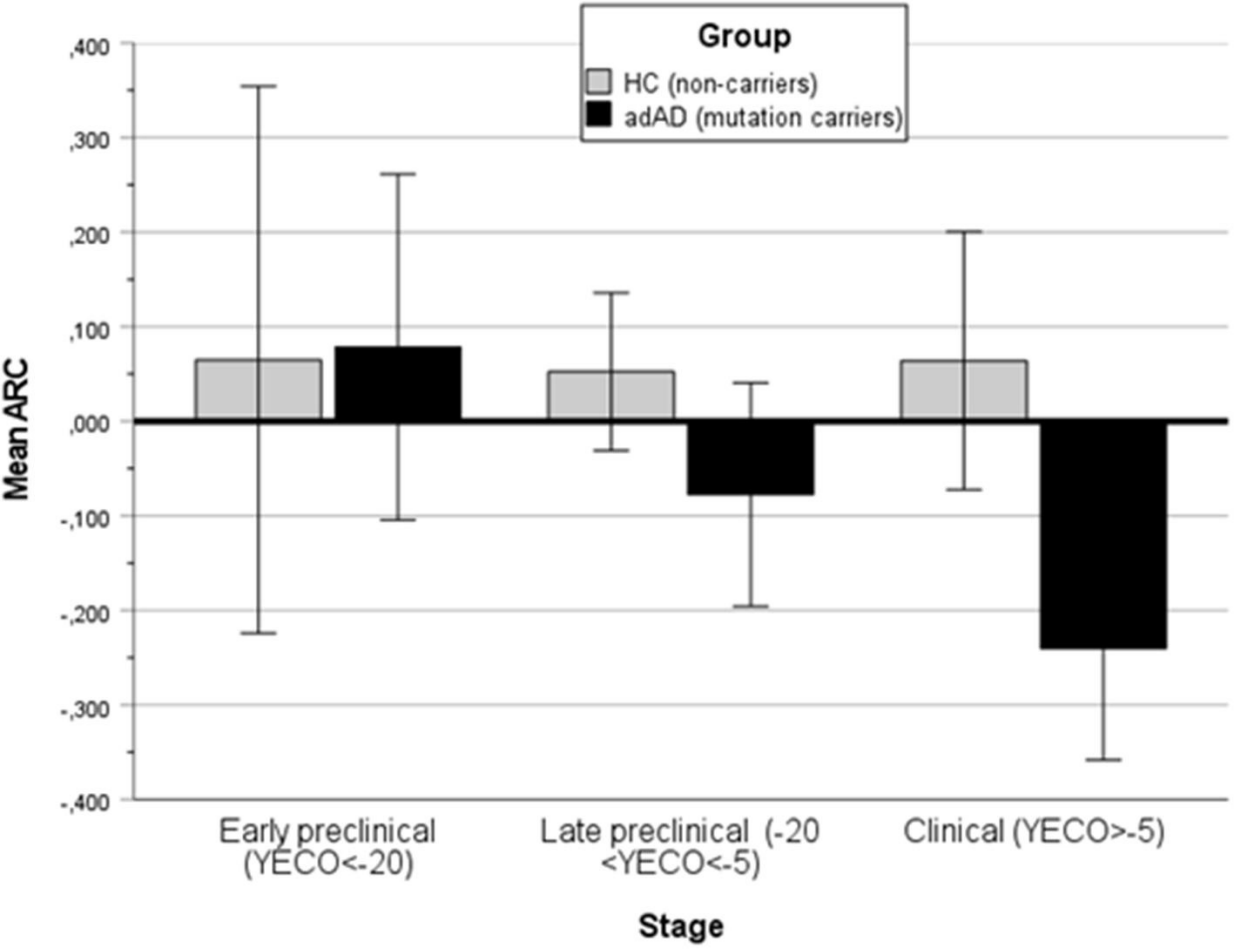
A bar graph showing the mean annual rate of change (ARC) in the HC and all AD groups (aAD, pAD, and dAD) in three stages of disease development: Early preclinical (YECO < −20), late preclinical (−20 < YECO < −5), and in the clinical stage around the estimated clinical onset.

Next, the relationship was analyzed in each of the six domains. The non-linear regression of ARC in each domain on time (YECO) is reported as LOcally WEighted Scatterplot Smoothing lines, see Supplementary Figures 1–6. For the HC group, the regression lines were practically linear and parallel to the X-axis and ARC was very close to 0 in all domains, although relatively small for the entire time course that was covered by the study, see Figure 3 and Supplementary Table 1. For the AD group, the mean ARC was positive in the very early preclinical stage (YECO < −20), but later the mean ARC turned into negative ARC values in all six domains that increased with time, see Figure 3 and Supplementary Table 1. The decline started early in the executive and episodic memory domains about 10 years before clinical onset. The decline in other domains began later and was relatively close to the clinical onset of YECO.

## Discussion

The study of PE with repeated cognitive assessments in mutation carriers and non-carriers from six families with autosomal-dominant Alzheimer's disease included mutation carriers varying in the stage of disease development in addition to healthy non-carriers. The carriers were diagnosed with Alzheimer's Disease (dAD), or prodromal AD expected to develop into dementia in the near future (pAD) or were lacking symptoms and regarded as asymptomatic although they will develop dementia in the distant future (aAD). All participants were examined at a memory clinic with a standardized protocol for patients with suspected dementia including a cognitive assessment with 12 tests covering six domains.

The first aim was to study PE measured as the annual rate of change (ARC) in cognition in the four groups (dAD, pAD, aAD, and HC). Results showed that PE aggregated across cognitive tests was positive in HC (M±SD: 0.056 ± 0.115), which is lower than reported in the previous literature (Van der Elst et al., 2008), probably depending on the length of the retest interval that was relatively long in this study (about 3 years in HC, aAD, and pAD, while it was about 1 year in dAD) compared short in many studies (Gross et al., 2018; Jutten et al., 2020; Samaroo et al., 2020). The hypothesis that mean ARC was equal to 0 was rejected in HC, but not in aAD implying that PE was absent or too small to be observed in aAD. The PE in HC was larger than in aAD individuals (M±SD: 0.007 ± 0.170), who lacked symptoms and were cognitively unimpaired despite carrying a mutation that will result in AD in the future. To speculate, the aAD individuals may have a subtle and unrecognized disturbance at this early stage about a decade prior to the estimated clinical onset. The results also showed that there was a negative PE in the dAD individuals, who were evaluated as mildly demented (MMSE M±SD: 21.0 ± 5.3) and the PE was lower than PE in the pAD group. This pattern of results supports that a practice effect exists in normal aging and is absent in clinically diagnosed AD as reported previously (Zehnder et al., 2007; Hassenstab et al., 2015; Elman et al., 2018; De Simone et al., 2021).

A few outliers in the mean ARC were observed. Two participants had extremely low mean ARC values (< -0.7, see Figure 1) and, in addition, they had short retest intervals that may have resulted in unreliable estimates that exaggerated the level of mean ARC. These mean ARC values are lower than the expected global cognitive decline (average across nine tests) previously estimated to be −0.43 in the mild stage of AD dementia (Almkvist and Bäckman, 1993). Finally, it should be pointed out that the negative ARC values represent values of annual progression of AD when practice effects are minor or absent.

The second aim was to study PE in specific cognitive tests with the expectation to find differences in correspondence with cognitive vulnerability associated with aging and disease (Cooper et al., 2004; Calamia et al., 2012; Salthouse, 2015). In order to improve stability across groups and tests, the pAD and dAD groups were combined into a symptomatic group and the 12 tests were aggregated into six domains (verbal, visuospatial, STM, executive, and attention). Now, the groups were differentiated in five of the six domains, and the effect of the domain was not significant, as well as the group-by-domain interaction. The largest power in differentiating the groups was obtained in the executive and attention domains that comprised timekeeping tests (Digit Symbol and TMTB as well as TMTA). This significant differentiation was obtained based on large negative and significant retest scores in the sAD group in executive and attention domains and not by positive PE in HC and/or aAD groups. In a similar vein, the preclinical decline in adAD in attention and executive function has recently been reported (Medina et al., 2021).

The significant and largest PE was observed in the verbal domain in the HC group in line with previous research (Calamia et al., 2012; Salthouse, 2015). PE was also positive in the verbal domain in the aAD group, although not significant. To speculate, the level of PE across cognitive domains in AD and HC is linked to cognitive vulnerability, i.e., lowest in the most vulnerable domains in AD considered to be episodic memory, executive, and visuospatial functions (Bateman et al., 2012; Almkvist et al., 2017). The largest PE was found in the verbal ability which is considered to be the least vulnerable domain in AD and in normal aging.

The third aim was to study the relationship between the size of PE and disease advancement estimated by YECO in the AD (mutation carriers with manifest and latent disease) and HC (healthy and cognitively unimpaired non-carriers). In the combined AD group, the relationship was linear and marked in the mean ARC. The change in mean ARC across time was about 0.06/year, which is less than the reported rate of change in previous research (Van der Elst et al., 2008). The low mean ARC in this study could be due to the long retest interval compared to the shorter retest intervals used in previous research (Falleti et al., 2006; Calamia et al., 2012; Machulda et al., 2013; Salthouse, 2015; Scharfen et al., 2018a). The type of test (screening vs. domain-specific) may impose variation in PE (Gross et al., 2018).

In the AD group, the mean ARC began to deviate from the mean level in the HC group about 20 years prior to the clinical onset and the confidence interval for the AD and HC groups occurred when YECO was 10–15 years ahead of the estimated clinical onset. The intersection of regression lines and confidence interval in the HC and AD groups in this study on PE are in agreement with reports of trajectories in cognitive tests using separate measures in AD (Bateman et al., 2012; Almkvist et al., 2017; Medina et al., 2021). The finding that aAD individuals did not demonstrate a significant PE or a significant difference compared to HC individuals when assessed about 20 years ahead of the clinical onset is a novel finding.

It was observed that the level of PE varied a lot, particularly in the early preclinical stage of disease in the aAD sub-group of AD. However, the number of individuals in this group is too few to analyze this finding further. One possibility may be to analyze the relationship between mean ARC and a biomarker in general by including all cases, both non-carrier and carriers.

The main body of recent research on PE has focused on PE with short retest intervals and PE as a marker of cognitive progression, while relatively few studies have focused on PE observed at long retest intervals as in this study. It has been suggested that the mechanism of PE is related to various learning and memory processes, e.g., remembering test items, answers, and problems related to explicit declarative learning and retrieval processes related to the test content (Gross et al., 2018; McDermott, 2021). In contrast, the PE results of this study obtained with long test intervals and a comprehensive cognitive assessment are suggested to be related to procedural learning and memory when performing cognitive tasks repeatedly. A similar suggestion was proposed (named as a context effect) in a recent study of MMSE with a short test interval (Gross et al., 2018). In theory, this memory has been described as implicit and keeping knowledge relatively intact across time. The division of learning and memory into explicit declarative and implicit procedural systems varying in learning mode (consciously vs. unconsciously) and retrieval mode (recollection vs. acting) was suggested years ago (Squire, 2004; Squire and Dede, 2015). To this end, a meta-analysis has shown that performance in procedural learning and memory tasks appears to be preserved in individuals with aMCI and AD dementia compared to healthy older adults (De Wit et al., 2021). The distinction of performance in declarative and procedural memory in AD was supported in a large study on MMSE in patients with AD with reduced episodic memory by a PE at retest 4 months later (Gross et al., 2018). Recently, it was demonstrated that patients with MCI and cognitively unimpaired adults did not differ in performance of the classical procedural learning task (mirror tracking), while groups differed in typical episodic memory (the RAVL test) (De Wit et al., 2022).

In addition, a number of general factors operate during testing the second time and later, for instance relief from factors that hamper individuals from optimal cognitive performance (uneasiness, concerns of being tested) and factors that may improve performance the second time (coping/adaption associated with the experience of testing, change in strategies how to solve tasks) (Lievens et al., 2007). A favorable feature of the present study that was the complete examination was a 2-day long visit, the tests were the same, the psychologist was the same, and personal was the same to a large extent over the years. Taken together, it is suggested that part of PE in the present study can be understood as an example of procedural learning and memory that promote performance in cognitive testing when repeated. Interestingly, the brain structures involved in procedural learning and memory are different from the structures involved in AD (De Wit et al., 2021).

This study is based on a relatively small sample of mutation carriers and non-carriers from six adAD families; this is a disadvantage that has to be kept in mind. Particularly, the small sample size was obvious in the pAD and dAD groups. The material was analyzed both in terms of group comparisons and in terms of regression analysis to find converging results that could strengthen the conclusions. The fact that Alzheimer's disease was studied in four groups defined on genetics from no disease in HC to the asymptomatic stage, across mild and finally marked cognitive impairment in AD represents a favorable and unique feature of this study in contrast to other studies with clinically defined disease stages (Calamia et al., 2012; Duff and Hammers, 2020; Jutten et al., 2020). It is also a favorable feature that the retest interval was long and that cognition was studied extensively with several tests from six cognitive domains. This made it possible to compare PE across cognitive domains in interaction with stages of AD development and in relation to the estimated remaining time to the clinical onset of AD.

There are some implications of the present findings for clinical application and research. If the expected practice effects of repeated cognitive testing were not considered, previous results in follow-up clinical examinations and longitudinal studies may need to be reinterpreted. Furthermore, clinical trials may have come to incorrect conclusions on the effects of treatment if the PE phenomena were not regarded. However, the size of PE and the influence of covariates on PE has to be established in future research before it could be used in research and clinical application. The potential benefit of absent PE in short retest intervals as a marker of cognitive decline in aging and mild disease has been well documented in previous research (Zehnder et al., 2007; Hassenstab et al., 2015; Elman et al., 2018; Jutten et al., 2020; Samaroo et al., 2020; De Simone et al., 2021). Finally, the mechanism of PE is not well understood. This fact makes it necessary to study both task-related cognitive factors as well as covert affective reactions.

To conclude, PE measured as ARC based on long retest intervals (about 3 years) were found in healthy and cognitively unimpaired middle-aged individuals (non-carriers from autosomal-dominant AD families) in age-insensitive cognitive domains. PE were also found in asymptomatic mutation carriers from AD families in the verbal cognitive domain when they were assessed long before the estimated clinical onset of AD. No PE, but a cognitive decline was obvious in symptomatic mutation carriers with mild cognitive impairment. In theory, PE are suggested to reflect that the person uses procedural learning and memory to master cognitive task demands in repeated testing.

## Data availability statement

The original contributions presented in the study are included in the article/Supplementary material, further inquiries can be directed to the corresponding author/s.

## Ethics statement

All participants were aware of their risk to develop AD. This information was given prior to the clinical examination. They also received genetic counseling in connection with the study and no one asked for information on their genetic status before the first visit or after the first visit. After the second follow-up examination, two asymptomatic participants opted for genetic testing after the completion of the examination. All subjects provided written informed consent to participate in the study. All examiners were blinded to the participants' mutation status. The study was approved by the Ethics Committee of Karolinska University Hospital at Huddinge and was conducted according to the Declaration of Helsinki and subsequent revisions.

## Author contributions

Both authors fulfill the ICMJE criteria for authorship. Other collaborators have been mentioned and thanked for their assistance in parts of this study. Both authors have read and agreed to the final version of the manuscript.

## Funding

This study was supported by grants from the Swedish Dementia foundation, Swedish Brain Foundation, Regional Agreement on Medical Training and Clinical Research (ALF) between Stockholm Region and Karolinska Institutet, Swedish Alzheimer Foundation, Stohnes Foundation, and Gamla Tjänarinnor Foundation.

## Conflict of interest

The authors declare that the research was conducted in the absence of any commercial or financial relationships that could be construed as a potential conflict of interest.

## Publisher's note

All claims expressed in this article are solely those of the authors and do not necessarily represent those of their affiliated organizations, or those of the publisher, the editors and the reviewers. Any product that may be evaluated in this article, or claim that may be made by its manufacturer, is not guaranteed or endorsed by the publisher.
