# Supplementary material for: Practice effects in cognitive assessments three years later in non-carriers but not in symptom-free mutation carriers of autosomal-dominant Alzheimer's disease: Exemplifying procedural learning and memory?
Source: Front Aging Neurosci. 2022 Oct 5;14:905329. doi: 10.3389/fnagi.2022.905329 (PMC9580215; doi:10.3389/fnagi.2022.905329)

Supplement Table 1. Results in 12 cognitive tests in z-score (M±SD) for the four groups at baseline (non-carriers, asymptomatic mutation carriers of adAD, prodromal adAD, and dementia adAD.

Non-carriers Mutation Carriers

Cognitive test HC aAD pAD dAD p η^2^

Information -1.30±1.14 -0.74±0.13 -1.21±1.01 -2.11±1.92 ns 0.09

Similarities -0.89±1.18 -0.12±0.91 -0.79±1.68 -2.33±1.61 ** 0.20

Block Design +0.57±1.35 +0.97±1.08 -0.26±0.91 -2.86±1.17 *** 0.40

Rey-O copy -0.26±2.04 -0.26±2.67 +0.28±0.19 -4.09±3.87 * 0.16

Digit Span -0.04±1.08 -0.10±0.67 +0.47±1.17 -1.19±1.46 ns 0.11

Corsi Span +0.64±1.31 +0.62±1.30 -0.45±1.16 -1.78±1.06 * 0.14

RAVL learning +0.14±0.84 +0.06±0.68 -1.74±0.40 -2.63±0.72 *** 0.56

RAVL retention -0.02±0.98 +0.10±0.89 -1.99±0.63 -2.63±0.40 *** 0.46

Rey-O retention +0.15±0.89 +0.37±0.79 -2.05±0.76 -2.44±0.30 *** 0.55

Digit Symbol +0.61±0.95 +0.66±0.88 -0.56±1.19 -2.28±0.95 *** 0.45

TMTA +0.72±0.64 +0.67±0.57 +0.15±0.86 -1.14±1.88 *** 0.29

TMTB +0.46±0.91 +0.53±0.42 -0.41±0.94 -3.08±2.97 *** 0.43

ns = not significant, * = p<0.05, ** = p<0.01, *** = p<0.001

Supplement Table 2. Practice effects expressed as the annual rate of change (ARC) across cognitive tests at baseline in non-carriers (Healthy Comparison group, HC) and mutation carriers varying in stage of AD disease course (asymptomatic, prodromal and demented). Significance and eta-square (η^2^) from one-way (group) ANOVA on each test.

Non-carriers Mutation Carriers

Cognitive test HC aAD pAD dAD p η^2^

Information +0.15±0.39 +0.07±0.18 +0.32±0.83 -0.39±0.46 * 0.15

Similarities +0.23±0.38 +0.11±0.25 -0.41±1.19 -0.19±0.46 * 0.16

Block Design +0.08±0.41 -0.03±0.44 -0.49±0.71 -0.23±0.81 ns 0.11

Rey-O copy -0.00±0.55 -0.20±1.29 -0.43±0.45 -0.69±0.65 ns 0.05

Digit Span +0.01±0.43 +0.08±0.25 -0.20±0.37 -0.47±0.25 * 0.13

Corsi Span -0.06±0.49 -0.12±0.15 +0.03±0.21 -0.43±0.40 ns 0.02

RAVL learning +0.11±0.35 +0.09±0.32 -0.09±0.39 -0.42±0.79 ns 0.11

RAVL retention +0.02±0.35 +0.18±0.47 -0.14±0.16 -0.44±0.38 * 0.11

Rey-O retention +0.11±0.42 -0.07±0.25 -0.11±0.27 -0.17±0.08 * 0.08

Digit Symbol +0.02±0.17 -0.00±0.18 -0.19±0.25 -0.35±0.32 ** 0.23

TMTA -0.02±0.40 +0.04±0.27 -0.54±0.85 -1.03±1.98 ** 0.19

TMTB +0.02±0.38 -0.08±0.17 -1.09±1.26 -0.26±0.51 *** 0.31

ns = not significant, * = p<0.05, ** = p<0.01, *** = p<0.001

Supplement Table 3. Practice effects expressed as the mean annual rate of change (ARC) over three years retest interval across the six cognitive domains in the early (YECO<-20) and late (-20<YECO<-5) preclinical stage and the clinical stage (YECO>-5) in the HC and AD groups. Significance and eta-square (η^2^) from one-way (group).

Preclinical stage Clinical stage

Group Early Late around onset p η^2^

HC (non-carriers) +0.06±0.06 +0.05±0.11 +0.06±0.16 ns 0.00

AD (mutation carriers) +0.08±0.14 -0.08±0.21 -0.24±0.36 p=0.10 0.17

ns = not significant, * = p<0.05, ** = p<0.01, *** = p<0.001

Supplement Figures

Figure 1


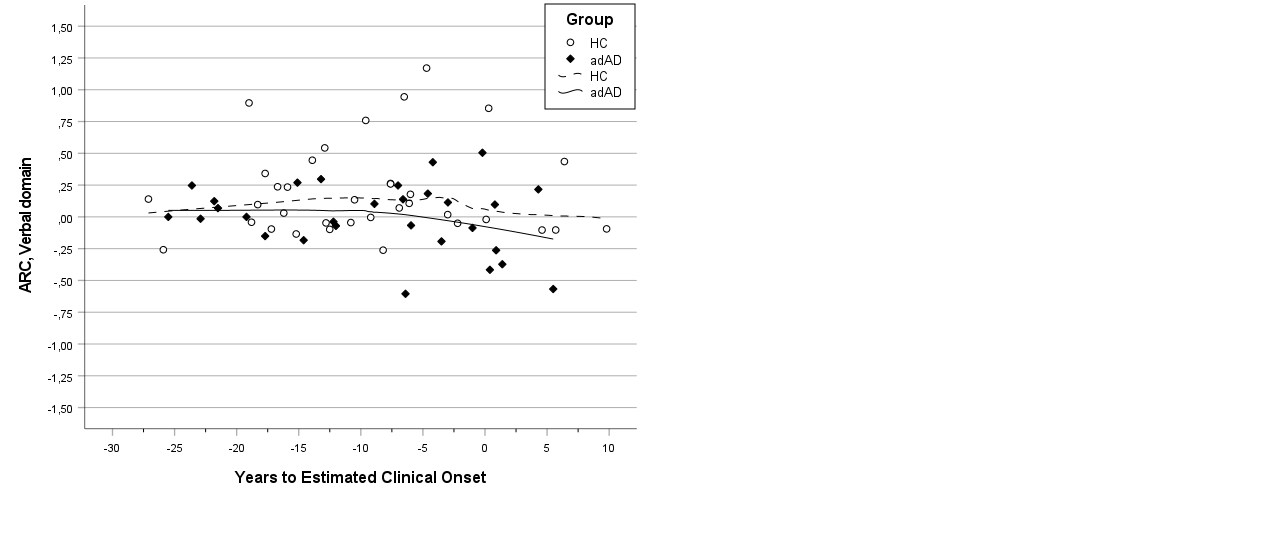


Supplement Figures

Figure 2


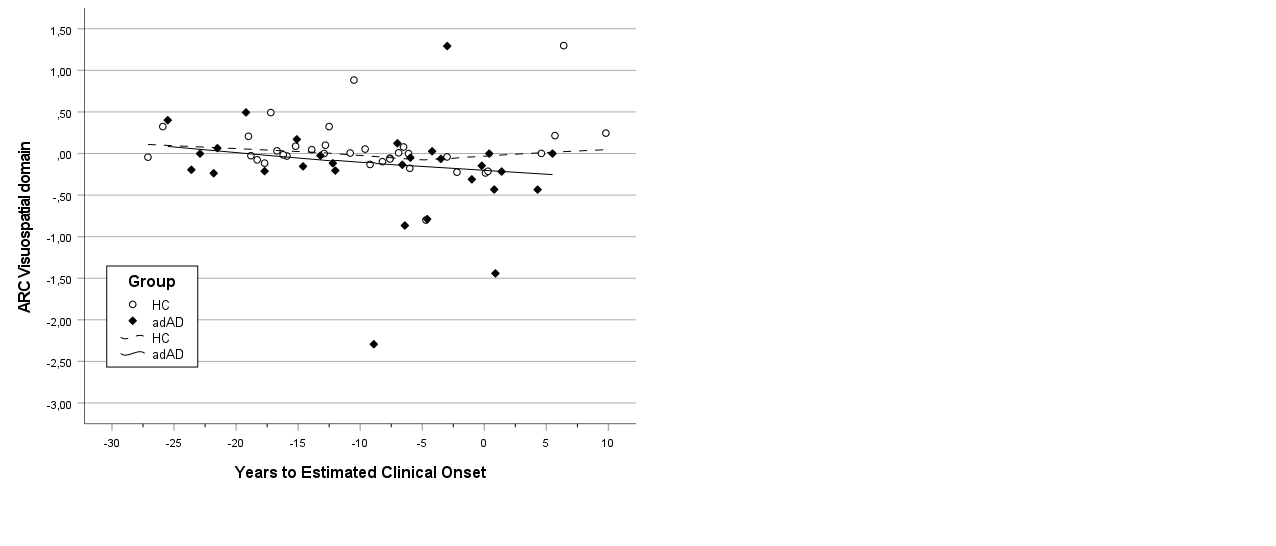


Supplement Figures

Figure 3


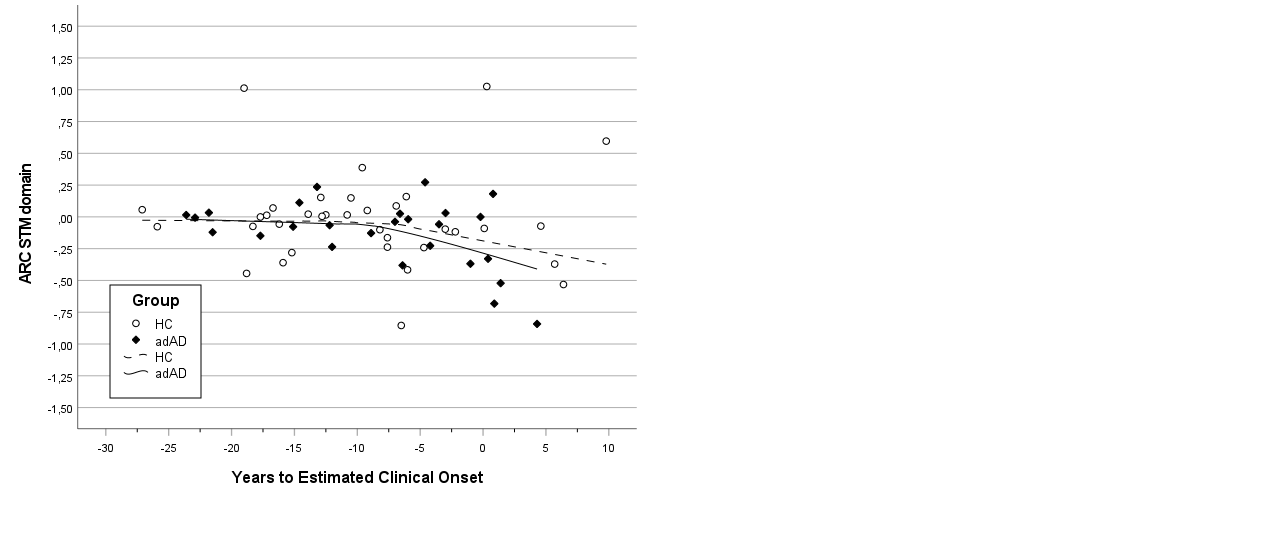


Supplement Figures

Figure 4


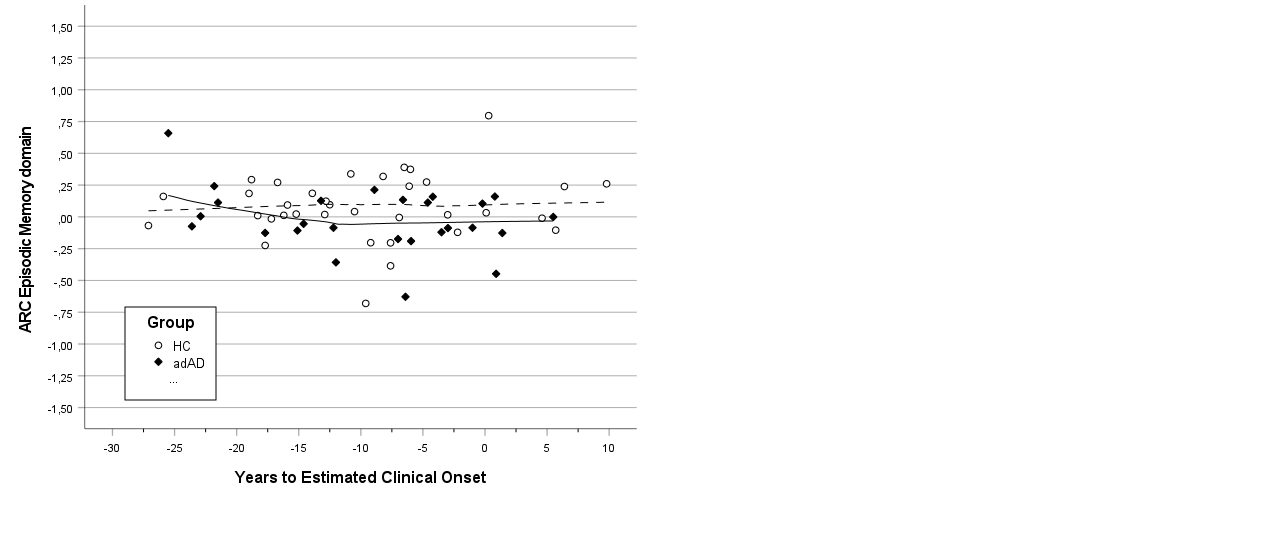


Supplement Figures

Figure 5


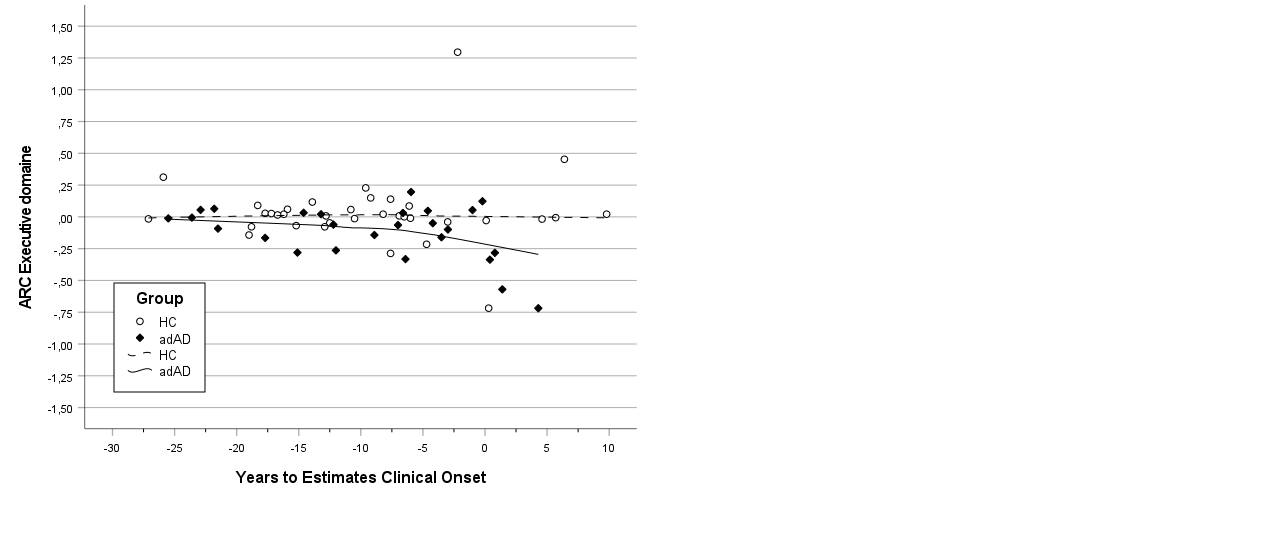


Supplement Figures

Figure 6


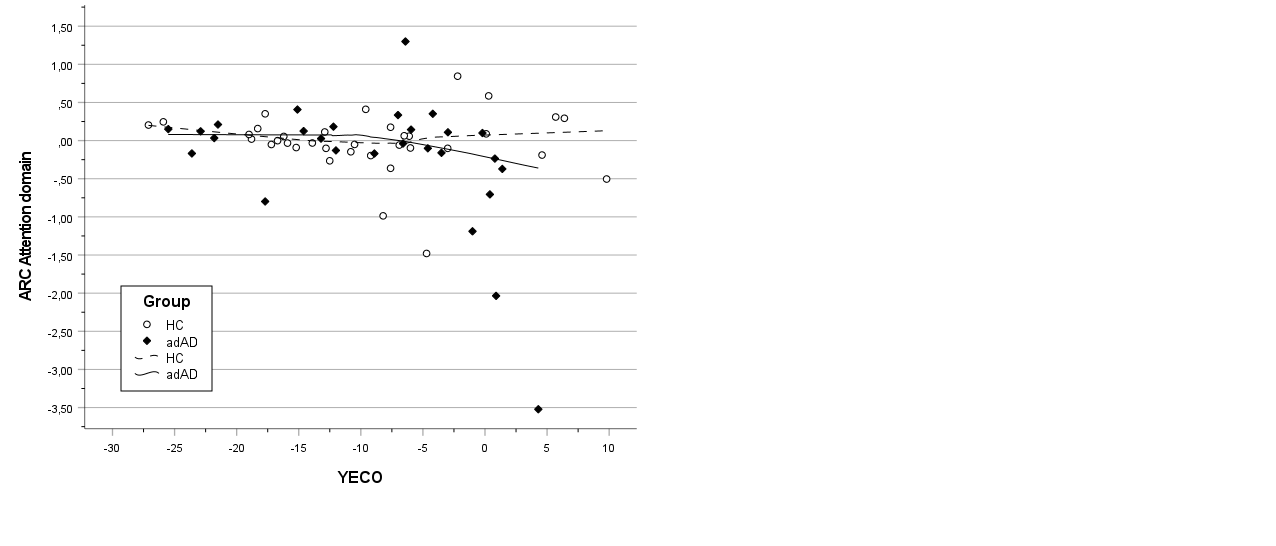

Supplement: Supplementary file 1 [file Data_Sheet_1.docx]
